# Transfer Learning Approaches for Neuroimaging Analysis: A Scoping Review

**DOI:** 10.3389/frai.2022.780405

**Published:** 2022-02-21

**Authors:** Zaniar Ardalan, Vignesh Subbian

**Affiliations:** ^1^Department of Systems and Industrial Engineering, College of Engineering, University of Arizona, Tucson, AZ, United States; ^2^Department of Biomedical Engineering, College of Engineering, University of Arizona, Tucson, AZ, United States

**Keywords:** neuroimaging, medical imaging, transfer learning, convolutional neural network, fine tuning, domain adaptation

## Abstract

Deep learning algorithms have been moderately successful in diagnoses of diseases by analyzing medical images especially through neuroimaging that is rich in annotated data. Transfer learning methods have demonstrated strong performance in tackling annotated data. It utilizes and transfers knowledge learned from a source domain to target domain even when the dataset is small. There are multiple approaches to transfer learning that result in a range of performance estimates in diagnosis, detection, and classification of clinical problems. Therefore, in this paper, we reviewed transfer learning approaches, their design attributes, and their applications to neuroimaging problems. We reviewed two main literature databases and included the most relevant studies using predefined inclusion criteria. Among 50 reviewed studies, more than half of them are on transfer learning for Alzheimer's disease. Brain mapping and brain tumor detection were second and third most discussed research problems, respectively. The most common source dataset for transfer learning was ImageNet, which is not a neuroimaging dataset. This suggests that the majority of studies preferred pre-trained models instead of training their own model on a neuroimaging dataset. Although, about one third of studies designed their own architecture, most studies used existing Convolutional Neural Network architectures. Magnetic Resonance Imaging was the most common imaging modality. In almost all studies, transfer learning contributed to better performance in diagnosis, classification, segmentation of different neuroimaging diseases and problems, than methods without transfer learning. Among different transfer learning approaches, fine-tuning all convolutional and fully-connected layers approach and freezing convolutional layers and fine-tuning fully-connected layers approach demonstrated superior performance in terms of accuracy. These recent transfer learning approaches not only show great performance but also require less computational resources and time.

## Introduction

Neuroimaging data provide a rich information source for clinicians to make decisions about diagnosis and treatment of different brain disorders. Utilizing advanced computational methods to analyze neuroimaging data, alongside physician's interpretation, can enable more accurate clinical decisions. These neuroimaging data include Magnetic Resonance Imaging (MRI), functional Magnetic Resonance Imaging (fMRI), Positron Emission Tomography (PET), and Electroencephalography (EEG). MRI, a non-invasive neuroimaging technology, utilizes a magnetic field to generate informative images of the brain (or any other tissue of subject's body). It produces detailed, three dimensional (3D) anatomical scans of the brain which can then be utilized in detection and diagnosis of diseases (Briani et al., 2013).

Functional Magnetic Resonance Imaging (fMRI) measures the dynamics of the blood flow to detect brain activities. When the neurons of an area of the brain is activated, the blood flow of that region of the brain will increase. Measuring blood flow results thus allows for measuring brain activities. fMRI is also non-invasive and produce four-dimensional data, three dimensions for depth, width, and height of the brain, and one dimension for temporal changes (Agosta et al., 2012). Positron Emission Tomography (PET) is a type of nuclear medicine procedure that measures the metabolic or biochemical function of the brain. PET is considered as a minimally invasive procedure (Lameka et al., 2016). PET scans are mostly used for detecting brain tumors. Malignant tumors in brain demonstrates changes in glucose metabolism and these changes can be detected using PET, the most common PET tracer. PET can also measure the most metabolically active target for stereotactic biopsy (Wong et al., 2002; Holzgreve et al., 2021).

Electroencephalography (EEG) measures the electrical activity in brain to detect abnormalities using electrodes which are often fixated on an EEG cap. Since there are no devices going inside subject's body, the EEG is categorized as non-invasive method. EEG data are usually a one-dimensional wave that can be processed to detect abnormalities in brain activities (Nagel, 2019).

Advances in deep learning for healthcare problems have resulted in development and evaluation of multiple algorithms in various areas such as diagnoses and prognoses of different neurological disorders (Khan et al., 2019; Wang et al., 2019). Among deep learning algorithms, Convolutional Neural Network (CNN) models have found important applications in areas including but not limited to tumor detection (Saba et al., 2020), Alzheimer's Disease (AD) diagnosis (Eitel et al., 2019), decoding brain behavior and activities (Gao et al., 2019a), and Parkinson's Disease (PD) (Choi et al., 2020). However, using CNN in neuroimaging is challenging and not straightforward. First, it requires a large amount of annotated training data, but there are relatively limited large, publicly-available neuroimaging datasets, in comparison to general imaging datasets. Second, even if large datasets are available for training, it is computationally expensive to train CNN networks from scratch (Khan et al., 2019).

**Figure 1:**
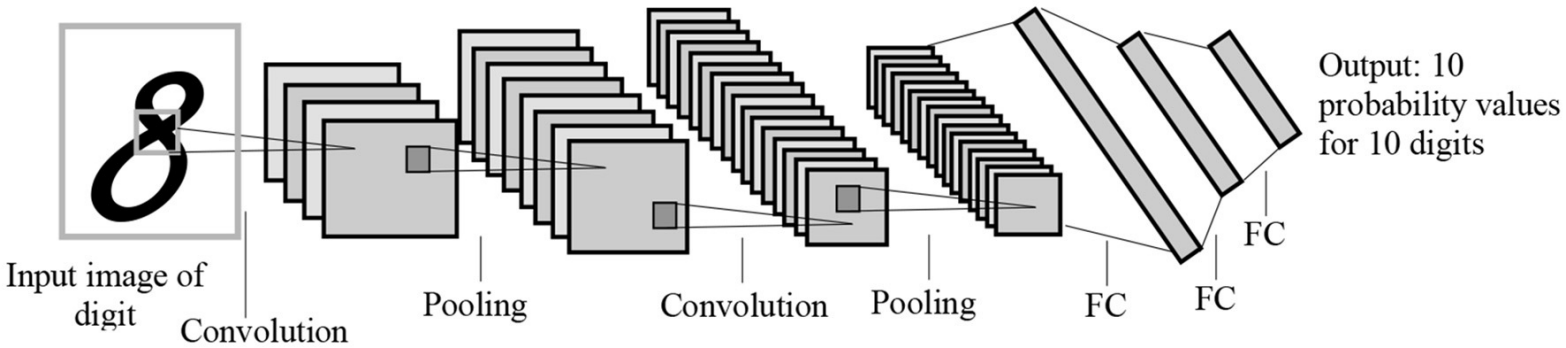
The LeNet architecture for letter recognition, one of the first CNN architectures for image processing. FC, Fully-connected layer. The architecture was designed for handwritten digit recognition.

To address these challenges, many studies have adopted transfer learning techniques, which allow for transferring learned features from one domain (source) to another domain (target). Since most transfer learning methods use CNN as a base model, we provide an example of how transfer learning can be implemented using CNN. CNN algorithms include convolutions, pooling, and fully-connected layers, with each layer learning different features. Figure 1 shows the LeNet CNN architecture with two convolution layers, two pooling layers, two fully-connected layers, and an output layer (Lecun et al., 1998).

In convolution layers, there are one or more convolution kernels or filters. The convolution kernels, using shared weights, learns image features such as detecting edges (the Laplacian edge detector), vertical lines (Sobel vertical line detect), and horizontal lines (Sobel horizontal line detector).

Consider a domain *D* with *X* as the feature space where *X* = {*x*_1_, *x*_2_, …, *x*_*n*_}. For example, given a specific domain *D* = {*X, P*(*X*)}, where *P(X)* is the marginal distribution function of *X*, and *T* = {*y, f*(*x*, Θ)} is the task with *y* as a set of labels and *f*(*x*, Θ) as the predictive model learned from domain *D by training* Θ, *the model parameters*. The *f*(*X*_*i*_) = ŷ_*i*_ is the predicted class for the *ith* data learned from training data. Model parameters Θ in imaging tasks are either convolution filters or weights of connections between fully-connected layers. For the convolution kernels, the kernel can be 2D with size set to 3 × 3, 5 × 5, and 7 × 7 matrix of weights in most of pre-trained architectures. These weights are initialized using initializers such as Glorot/Xavier uniform initializer (see Equation 1), He normal initializer, or random normal initializer.

(1) $$\begin{array}{r} \text{Glorot range:}\left[\frac{-1}{\sqrt{inUnits}},\frac{1}{\sqrt{inUnits}}\right] \end{array}$$

For an image classification task, consider *x*_*i*_ as the *ith* image and dimensionality of 2D or 3D. For 2D images, each pixel is denoted by *i* and *j*, representing row and column of the pixels and *X* is the collection of all available images. Here, let us define H as the convolution filter and *H*[*u, v*] be the value of the convolution filter at row *u* and column *v*. Also, *F[i*+*v,j*+*v]* is the corresponding value of the image at the row *i*+*v* and column *j*+*u*. Therefore, the output pixel of convolution operation at the pixel *i, j* is calculated as follows:

(2) $$\begin{array}{r} G\left[i,j\right]=\overset{k}{\underset{u=-k}{\sum }}\overset{k}{\underset{v=-k}{\sum }}H\left[u,v\right]F\left[i+u,j+v\right]\;\left(2\right) \end{array}$$

where *inUnit* is the number of units in the input matrix. Then after initialization, the algorithm can be trained on a specific problem so that each kernel would detect a feature in the CNN network. After convolution operation, there is an activation function, which for most CNN algorithms it is Rectified Linear Units (ReLU). The ReLU is calculated using Equation (3).

(3) $$\begin{array}{r} \text{ReLU(x) = max (0, x)} \end{array}$$

CNN starts with a training dataset of D= ${\left\{\left(x_{i},y_{i}\right)\right\}}_{i=1}^{N}$, where N is the size of training dataset, *x*_*i*_ is the features of the *ith* data and *y*_*i*_ is the gold label of the data. The CNN learns the *f*(*x*_*i*_, Θ), where Θ denotes model parameters and the *f* is the prediction function. The goal of the CNN is to minimize the loss function of the model so that L is minimized:

(4) $$\begin{array}{r} L=\frac{1}{N}\overset{N}{\underset{i=0}{\sum }}L\left(f\left(x_{i},\Theta \right),\;y_{i}\right) \end{array}$$

Most binary classification CNN algorithms use cross-entropy loss function (see Equation 5).

(5) $$\begin{array}{r} L\left({ŷ}_{i},y_{i}\right)=-\frac{1}{N}\overset{N}{\underset{i=0}{\sum }}\left[y_{i}\log{ŷ}_{i}+\left(1-y_{i}\right)\log\left(1-{ŷ}_{i}\right)\right] \end{array}$$

**Figure 2:**
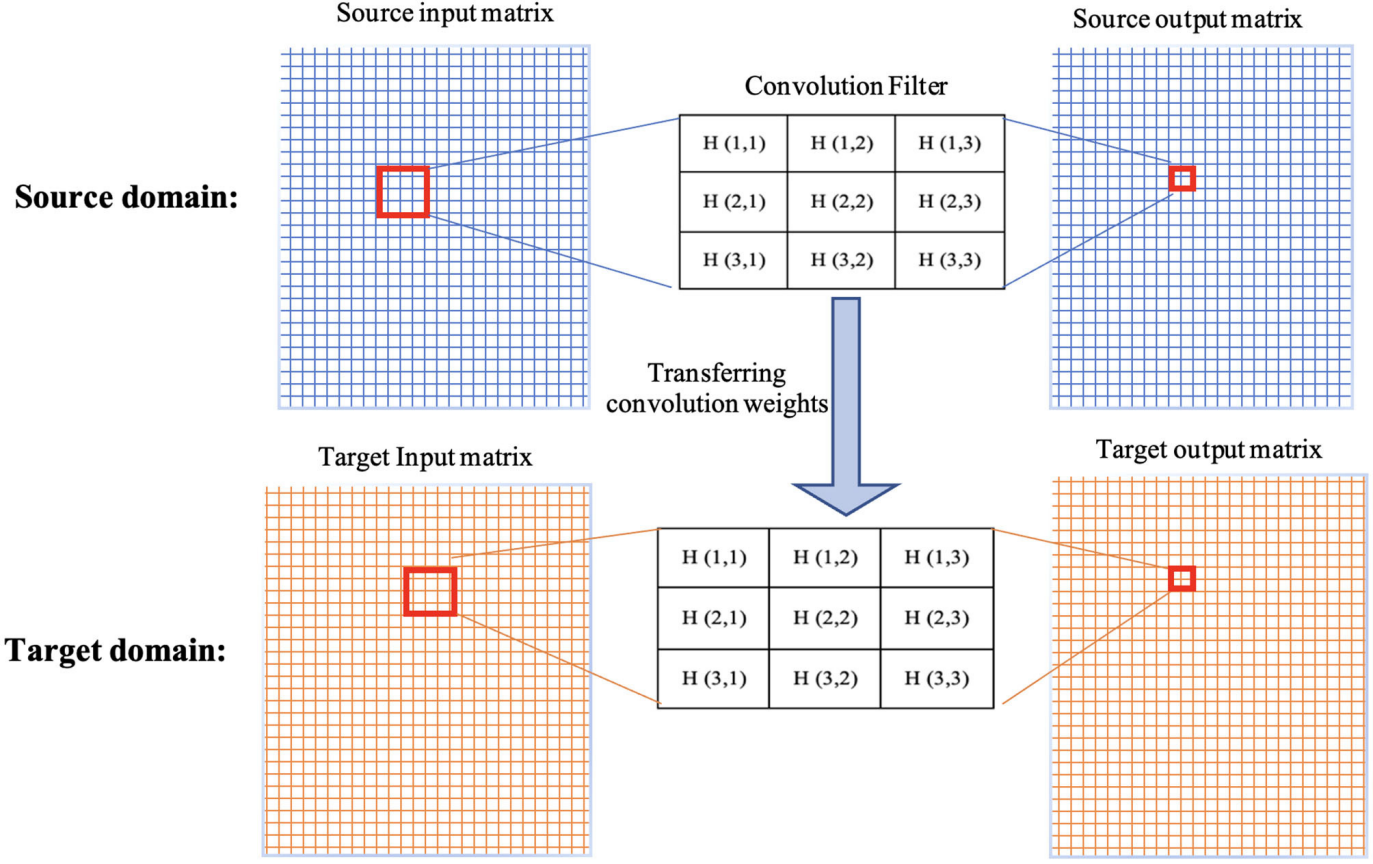
Demonstration of transferring weights of a convolution filter from source domain to target domain.

The weights/parameters of the model are optimized using algorithms such as stochastic gradient descent and Adam optimizer. The first layers in the architecture are more responsible for learning low-level image features such as lines, curves, edges, and their combinations. The latter layers are more responsible for high-level features to detect bigger pieces of an image such as tumors. Transfer learning is a method that transfer parameters (weights of convolution kernels and fully-connected connections) of a model trained on a dataset (source dataset) to the same model on another dataset (target dataset). This means when training a model on the target dataset for a given problem, instead of initializing parameters from a random procedure, pretrained parameters and weights are used (see Figure 2). Since different problems would share common features, transfer learning helps by starting from weights that can detect some useful features (such as the edge detector) instead of starting from random weights. In transfer learning, when some layers are frozen, the weights of those layers and their corresponding kernels are fixed. Fine-tuning means the model starts from these points as initialization for the kernel weights. Full training (i.e., training from scratch) means these weights will be initialized randomly. In transfer learning, some of these layers can be frozen, eliminating the need for training these layers and saving large amount of time and resources in training these models from scratch. Other non-frozen layers can then be modified to train the network based on the target dataset.

Another approach to perform transfer learning is to freeze all layers from source and add and train new layers. In addition, trained parameters from the source dataset can be used as initialized parameters for the target dataset. In this case, the whole network can be trained, allowing for the algorithm to converge faster, without the need for a huge number of epochs (iterations) to train.

There are several other approaches of transfer learning, each with their own cons and pros. Therefore, the aim of this paper is to perform a review of different transfer learning techniques used in neuroimaging and analyze the approaches, design characteristics, benefits, and drawbacks. Key considerations for the review are:

1. Type of transfer learning approach.
2. Performance of each approach.
3. Different datasets and modalities used for source and target datasets.
4. Neuroimaging research area of each study.

In one systematic review on neuroimaging data, Agarwal et al. (Agarwal et al., 2021) reviewed the literature transfer learning on only AD related problems. The main difference between this systematic review and our study is that the main focus of the Agarwal et al. (Agarwal et al., 2021) was on AD, while our current study reviews all neuroimaging related work. In another review, Buchlak et al. (Buchlak et al., 2021) reviewed the machine learning applications for glioma detection and some transfer learning approaches were discussed in their review but their main focus was on broader machine learning approaches. The main contribution of this paper is to help readers identify an appropriate approach of transfer learning for different tasks in the neuroimaging domain.

## Methods

We explored literature related to transfer learning in neuroimaging from January 2010 to December 31, 2021. The search was conducted on two databases, Scopus® and PubMed®, using the following keywords: neuroimaging and transfer learning. All papers including journal and conference proceedings were considered in the first round of title and abstract screening. For duplicates studies in both databases, only one was included and the other one was removed. If studies were not related to both neuroimaging and transfer learning, they were excluded in the screening phase. Full text articles from the screening phase were then further reviewed using the following inclusion criteria:

- Focuses on imaging problems such as classification, segmentation, or regression related to neurological conditions including AD, brain tumors, Multiple Sclerosis (MS), and PD.
- Uses neuroimaging data including Magnetic Resonance Imaging (MRI), Functional MRI (fMRI), or Positron Emission Tomography (PET).
- Uses machine learning techniques including traditional techniques such as support vector machine (SVM) or deep neural network algorithms such as CNN.
- Includes at least one performance metric such as accuracy, sensitivity, specificity, and area under receiver operating curve (AUC).
- Uses transfer learning techniques.
- Includes transfer learning implementation details.
- Includes details on source and target datasets.

From the included studies, following data were extracted:

1. Imaging type: Type of the imaging (such as: MRI, fMRI, and PET) used by studies for both source and target datasets were extracted for all studies. Some studies used different datasets for source and target domains or used multiple imaging type in one domain. In these cases, all used imaging types have been considered.
2. Datasets used for source and target.
3. Different types of machine learning algorithms: Whenever multiple algorithms were used by a study; all algorithms were extracted.
4. Neuroimaging research problems such as AD related diseases, brain tumors, and MS addressed by studies.
5. Transfer learning methods implemented by the literature were also another data that have been extracted. If multiple methods have been studied by one paper, all methods have been considered and discussed.
6. Performance metrics such as accuracy, sensitivity, specificity, and AUC.

**Figure 3:**
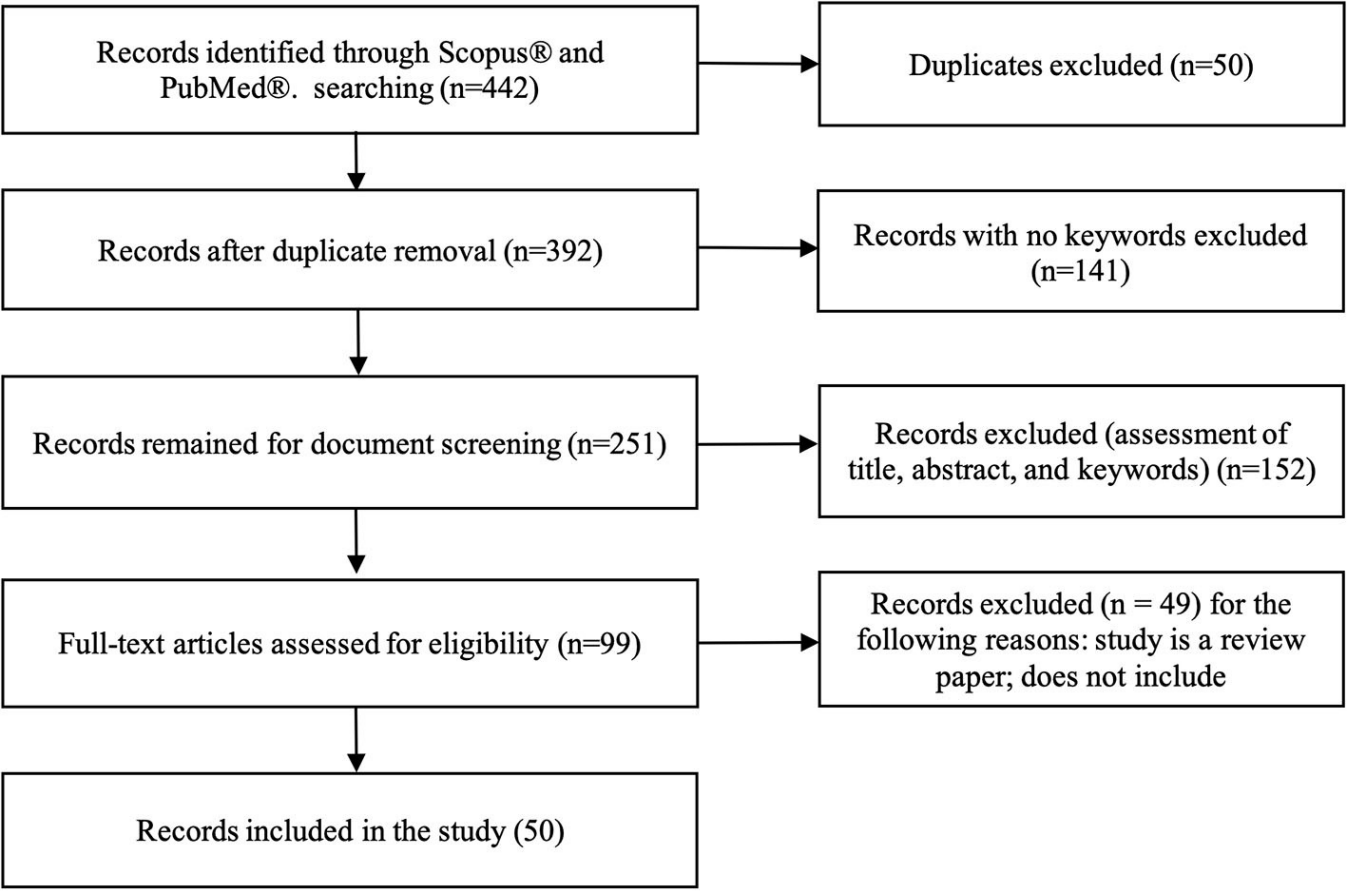
Flowchart illustrating literature search process and extraction of studies meeting the scoping review inclusion criteria.

A total of 422 studies were identified using both databases, Scopus® and PubMed®. After removing duplicates, 392 studies were considered. Among them, 141 studies were excluded because there were no predefined keywords (transfer learning, neuroimaging) in the entire context of the study, leaving 251 studies were left for screening. Title and abstract screening resulted in 99 studies. After reading the full-text, 49 studies were excluded because they were either review papers, not transfer learning in the context of machine learning but psychological transfer learning, or they did not implement transfer learning, but only mentioned it in span of the paper. Finally, 50 studies were included for analyses (see Figure 3).

The rest of the paper is organized as follows: detailed review of the literature based on the four review considerations are explored in Section Results. In Section Discussion, main highlights of the literature are discussed, along with research directions and open questions in transfer learning. Section Conclusion provides a summary of the review.

## Results

Based on the inclusion criteria for the study, 50 studies were identified for review and analyses. Nine major categories of clinical problems were covered in these studies including AD detection, brain mapping, brain tumor detection, and MS. Most of the studies focused on AD related problems such as Mild Cognitive Impairment (MCI) detection likely because of availability of large datasets for AD such as the Alzheimer's Disease Neuroimaging Initiative (ADNI) dataset (Saykin et al., 2010). Brain mapping was also discussed by multiple studies.

### Source and Target Datasets

A diverse range of datasets were used for both source and target datasets. For the source datasets, 16 different datasets were used, of which ImageNet and ADNI were the most frequently used (19 and 17 times, respectively). Table 1 shows frequency of each dataset in source domain. In terms of imaging modality, MRI was the most used imaging data for the source domain. Natural images, all from ImageNet dataset, were the second most common source data, followed by EEG and fMRI. Table 2 shows data type combinations for source datasets.

**Table 1:** Frequency of datasets in source and target domains.

**Name of the dataset**	**Source**	**Target**
ImageNet	19	0
Alzheimer's Disease neuroimaging initiative (ADNI)	17	26
Human Connectome Project (HCP)	1	3
Brain Tumor Segmentation (BraTS)	1	2
The Open Access Series of Imaging Studies (OASIS)	1	3
Nathan Kline Institute-Rockland Sample (NKI-RS) Nooner et al., 2012	0	1
The National Alliance for Medical Imaging Computing (NAMIC)^a^	1	1
Kirby^b^	1	1
Rotterdam Scan Study (RSS)	1	1
MRBrains^c^	1	1
Internet Brain Segmentation Repository (IBSR)^d^	1	1
MS Lesions^d^	1	1
Brain-computer interface (BCI) Blankertz et al., 2004	0	1
High gamma dataset (HGD)^e^	0	1
Private datasets	9	12
WHO grade status	0	1
MS dataset	0	1
The Cancer Genome Atlas (TCGA)^e^	0	1
Harvard The Whole Brain Atlas (AANLIB)	0	1
Autism Brain Imaging Data Exchange (ABIDE) Li L et al., 2021	1	2
UK Bio-Bank (UKBB)	0	1
An Asian Cohort Saba et al., 2020	0	1
National Alzheimer's Coordinating Center (NACC)^f^	0	1
Hammers Adult Atlases (HAA)	1	0
Multi-Atlas Labeling Challenge (MALC)^g^	1	0
Functional connectivity dataset	1	0

a *MIDAS—Collection NAMIC: Public Data*.

b *Databases | Kennedy Krieger Institute*.

c *MRBrainS13 | Evaluation framework for MR Brain Image Segmentation*.

d *NITRC: Longitudinal Multiple Sclerosis Lesion Imaging Archive: Tool/Resource Info*.

e *The Cancer Genome Atlas Program—National Cancer Institute*.

f *NACC Researcher home page, NACC, Alzheimer's disease research, FTLD, NIA/NIH, database, neuropathology*.

g *2012 MICCAI Multi-Atlas Labeling Challenge Data*.

**Table 2:** Data types for source and target domains.

**Source domain**		**Target domain**	
**Data type**	**Frequency**	**Data type**	**Frequency**
MRI	31	MRI	36
Natural Images	18	PET	4
fMRI	5	fMRI	8
EEG	4	EEG	2
PET	3	CSF	1
Cerebrospinal fluid (CSF)	1		

For the target domain, 22 different datasets were utilized with ADNI, Open Accessible Summaries In Language Studies (OASIS) (LaMontagne et al., 2019), and Human Connectome Project (HCP) being the most frequently used datasets (ADNI 26, OASIS 3, and HCP 3 times, respectively). Brain Tumor Segmentation (BraTS) and Autism Brain Imaging Data Exchange (ABIDE) datasets were used twice, and the rest of datasets were explored by at least one study. Table 2 shows the different target datasets with their frequency. MRI and fMRI were the most frequently explored modality in target datasets, see Table 1.

### Algorithms

#### CNN-Based Algorithms for Transfer Learning

Sixteen studies designed their own custom CNN architecture (Han, 2017; Li H et al., 2018; Dai et al., 2019; Eitel et al., 2019; Oh et al., 2019; Pham et al., 2019; Thomas et al., 2019; Wee et al., 2019; Wu et al., 2019; Choi et al., 2020; Liu et al., 2022). For example, Eitel et al. (2019), utilized a 3D CNN consisting of four convolution layers followed by three pooling layers after first, second and fourth convolution layers. With a kernel size of 3 × 3 × 3, the model used exponential linear units for activation function and sigmoid function in the output layer for the classification. Furthermore, to reduce the overfitting dropout were applied. The model was pre-trained on ADNI data to separate AD from normal controls, and then fine-tuned on the MS dataset to separate MS patients from healthy controls. In another study, Choi et al. (2020) designed their own 3D CNN architecture consisting of five convolution layers followed by one pooling layer with a kernel size of 5 × 5 × 5 for all layers and ReLU activation. PET images of AD subjects and normal controls were used for training and then the weights were transferred to a Parkinson's Disease dataset. This suggests that designing new custom CNN would require training their architectures on a dataset on their own rather than to using publicly-available pre-trained models. Kalmady et al. (Kalmady et al., 2021) presented a cross-diagnosis transfer learning approach for obsessive-compulsive disorder detection using fMRI images from 188 cases vs. 200 normal controls. The input images were fed to a custom CNN+RNN architecture. A portion of their dataset was used as source and the rest as target dataset.

More than 70% of the studies utilized existing competitive algorithms such as VGG (Simonyan and Zisserman, 2015), AlexNet (Krizhevsky et al., 2017), ResNet (He et al., 2016; Ni et al., 2021), Inception (Szegedy et al., 2015; Liu et al., 2021). VGG was the most commonly used algorithm among existing algorithms (excluding custom CNNs) mainly because VGG is already pre-trained on a large-scale dataset (ImageNet) and had strong performance on different problems including medical image processing (Gao et al., 2019a). The VGG16 consist of 13 convolution, 5 pooling, and 3 FC layers. The main difference between this network and other CNN architectures is that it uses deeper network with smaller convolution filters of size 3 × 3. This helped to gain significant improvement compared to other CNN networks (Simonyan and Zisserman, 2015).

**Figure 4:**
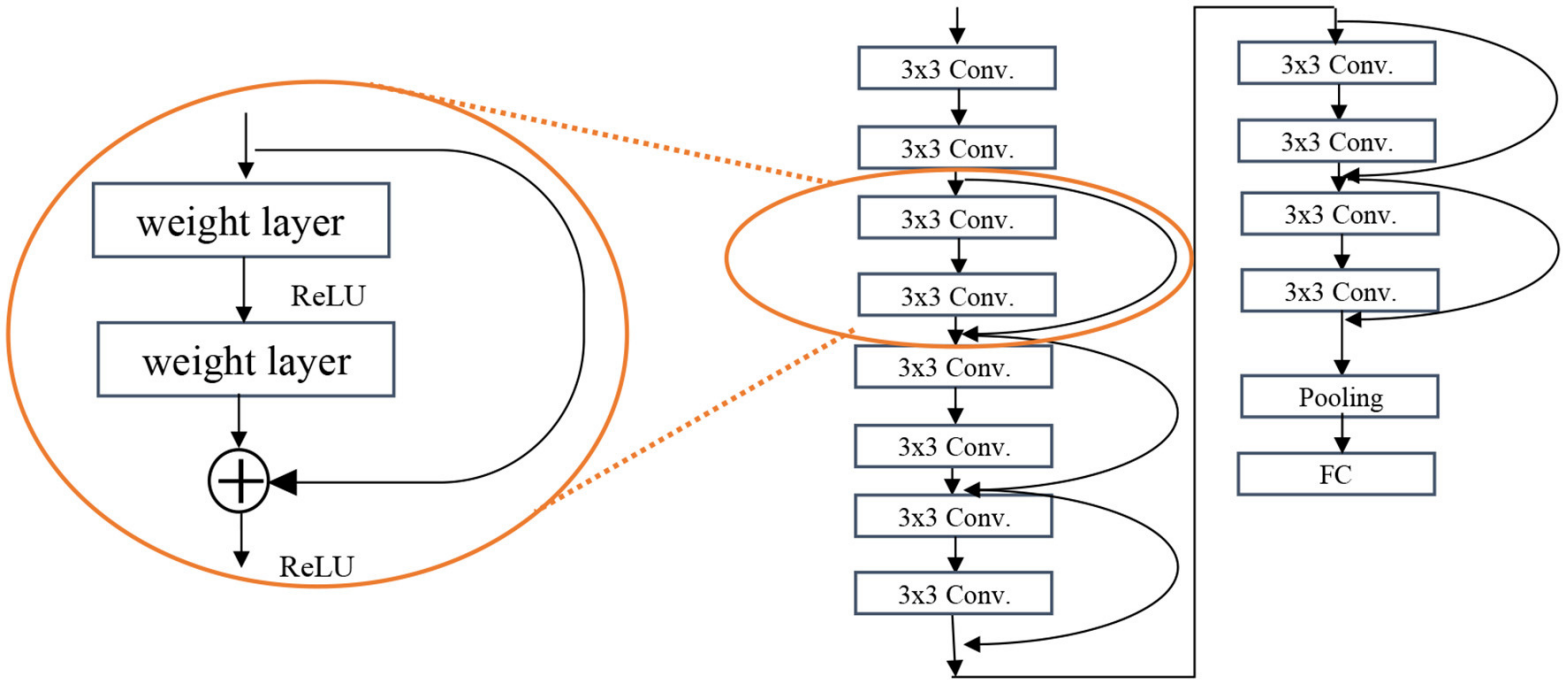
Residual block of the ResNet algorithm (left) and the ResNet 12 architecture (right).

The ResNet architecture is the second most used architecture. Deeper networks such as VGG are exposed to degradation problem and the accuracy gets saturated and then degrades rapidly. In ResNet, there is another element called residual block (see Figure 4) which takes the input of a layer and adds to the output (*f(x)* + *x*), called short connections. Short connections help solve the problem of degradation. The ResNet architecture consists of consecutive residual blocks, a pooling layer, and the output layer. Figure 4 shows the ResNet 12.

**Figure 5:**
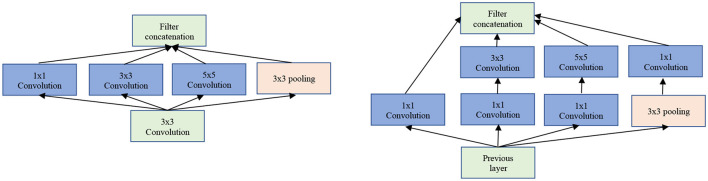
The GoogLeNet inception modules. Left: Naïve version of inception module. Right: Inception module with dimensionality reduction.

The Inception/GoogLeNet was the second (tied with ResNet) most used architectures. The GoogLeNet is an architecture stacked up using inception modules which are blocks consisting of multiple convolution and pooling layers (see Figure 5). It starts with Inception modules only at higher layers while keeping the lower layers in traditional convolutional way.

**Figure 6:**
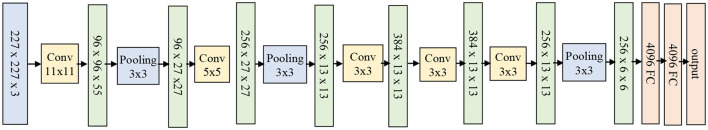
The AlexNet architecture.

The next most used architecture is AlexNet which is much deeper than LeNet but uses almost all elements of LeNet architecture. AlexNet contains eight layers: five convolutional layers and three fully-connected layers. AlexNet replaced *tanh* activation function with ReLU for the first time, which reduced the training time (six times faster on ImageNet dataset original implementation) and provided better performance. It also uses overlapping pooling layers that reduces the overfitting. The AlexNet architecture is shown in Figure 6.

Mehmood et al. (Mehmood, 2021) utilized MRI images from ADNI dataset to detect MCI subjects. A pretrained VGG-19 architecture was adopted to implement layer-wise transfer learning. The gray tissue segmentation was used to only focus on gray tissue when detecting MCI. The first 16 layers of VGG-19 which contains convolution and pooling layer were fixed and the last three layers were modified to account for new data. Two transfer learning settings were examined. In the first setting, eight convolution layers and three pooling layers were frozen. In the second setting, twelve convolution layers and four pooling layers were frozen. The second approach achieved 95.3% of accuracy (with 94% sensitivity and 96% specificity) and first approach achieved 93.8% accuracy on normal control vs. AD. For other classification tasks such as normal control vs. MCI and MCI vs. AD, the second approach performed better as well.

Kang et al. (2021) developed an ensemble model for AD diagnosis using a multi-model and multi-slice architecture. VGG16 and ResNet50 were slightly modified, and majority voting scheme was utilized on the merge of the multi-slice output. All slices were included in the VGG16 and pretrained on ImageNet dataset, with the first four convolution layers frozen, and the rest fine-tuned for the target dataset which includes more than 700 subjects of AD, MCI, and normal controls from ADNI. Bae et al. (Bae et al., 2021) studied AD vs. MCI classification task on ADNI dataset. MRI scans of 3,490 subjects from ADNI were included for training the models on source dataset and 450 MRI scans from ADNI were included for target dataset. ResNet50 was modified to decrease the number of trainable parameters from 23 to 4 millions by making residual blocks smaller and decreasing the number of channels at each layer. The training weights of source dataset were transferred and retrained on target dataset entirely.

Khan et al. (2019) selected VGG19 from ImageNet because it has the capability to adapt to different image classification tasks. This study implemented 2D convolution filters with the 3 × 3 size with a single stride for the entire network to ensure overlapping receptive fields to capture more information. Pooling filter size was 2 × 2 and stride 2. ReLU activation function was used for all hidden layers. A 2D CNN algorithm was implemented with 8, 16, and 32 slices out of 256 slices using an image entropy formulation instead of random selection of slices. Wang et al. (2019) used the pre-trained model of AlexNet to initialize their parameters and trained the whole model again. New layers were added to the end of AlexNet and trained from scratch. ReLU was used as an activation function in hidden layers instead of sigmoid function to prevent models from vanishing gradient issues. Local response normalization was used to help with generalization. To modify the structure of AlexNet for their problem, the FC layers were revised. In AlexNet, there are 1,000 classes but here there are two classes. Therefore, the last layer was replaced with a layer with only two classes. Simon et al. (2019) used AlexNet, ResNet-18, and GoogLeNet to implement transfer learning for classification of normal control, early MCI, MCI, late MCI, and AD on fMRI images from ADNI dataset. Images were resized to the size required by architectures. Weights were fine-tuned from the source dataset to ADNI. The results show AlexNet had better performance in terms of accuracy than others.

### Traditional Algorithms for Transfer Learning

Support Vector Machine (SVM) is a supervised learning classifier that finds the decision boundary with maximum margin for a given problem. SVM is effective in high dimensional space where the number of dimensions is greater than the number of samples. It is also memory efficient and uses different kernel functions to model different spaces. Therefore, SVM was used to classify neuroimaging data and to improve the results of transfer learning in several studies (Cheng et al., 2017; He et al., 2018; Van Opbroek et al., 2019; Buchlak et al., 2021). Cheng et al. (Cheng et al., 2021) presented a multi-auxiliary domain transfer learning approach for diagnosis of MCI subjects from ADNI dataset for both source and target datasets. MRI images were preprocessed and concatenated with cerebrospinal fluid features without transfer learning by more than 10% in almost all performance metrics including accuracy, sensitivity, and specificity. Kernel learning methods were used to transfer learned knowledge from one domain to another. Schwartz et al. (2012) used both SVM and logistic regression to help generalize the power of their method in transfer learning. Long Short-Term Memory (LSTM) algorithm was utilized to deal with the time dimension of 4D images in Human Connectome Project dataset. All other three dimensions were explored by a CNN architecture (Thomas et al., 2019). Their algorithm consisted of three main components, a 12-layer CNN feature extractor, a bi-directional LSTM unit, and a SoftMax output layer. Adaboost (Zhou et al., 2018), Connectome CNN (Al Vakli et al., 2018), DenseNet (Liang et al., 2018), U-Net (Dai et al., 2019), and SqueezeNet (Ebrahimighahnavieh et al., 2020) have been implemented by researchers (see Table 3).

**Table 3:** Different algorithms used for neuroimaging problems and their usage frequency.

**Task**	**Frequency (%)**	**Algorithms**	**Frequency (%)**
Classification algorithms	66 (91%)	VGG	11 (15%)
		AlexNet	8 (11%)
		ResNet	9 (13%)
		Inception/GoogLeNet	9 (13%)
		SVM	7 (10%)
		Custom CNN	14 (19%)
		SqueezeNet	1 (1%)
		ConnectomeCNN	1 (1%)
		DenseNet	1 (1%)
		Logistic Regression	1 (1%)
		TrAdaboost	1 (1%)
		Lasso	1 (1%)
		LSTM	2 (3%)
Segmentation	6 (9%)	U-Net	4 (6%)
		Custom CNN	2 (3%)
		**Total**	**72**

Most of the studies were focused on classification tasks and implementing classification algorithms. However, segmentation (Amin et al., 2019; Saba et al., 2020), regression (Schwartz et al., 2012; Dong et al., 2019), image translation (Han, 2017), and image annotation (Dai et al., 2019) were other tasks pursued in the literature (see Table 3). Moreover, most of algorithms utilized for transfer learning are deep learning methods which need extensive training (see Table 4).

**Table 4:** Machine learning vs. deep learning algorithms used for neuroimaging problems and their usage frequency.

**Type**	**Algorithms**	**Frequency (%)**
Deep learning 62 (86%)	VGG	11 (15%)
	AlexNet	8 (11%)
	ResNet	9 (13%)
	Inception/GoogLeNet	9 (13%)
	U-Net	4 (6%)
	Custom CNN	16 (22%)
	SqueezeNet	1 (1%)
	ConnectomeCNN	1 (1%)
	DenseNet	1 (1%)
	LSTM	2 (3%)
Machine leaning 10 (14%)	Lasso	1 (1%)
	SVM	7 (10%)
	Logistic Regression	1 (1%)
	TrAdaboost	1 (1%)
	**Total**	**72**

### Transfer Learning Approaches

There are different strategies in transfer learning in terms of how the layers are transferred (e.g., directly, fine-tuned, or reinitialized) (Kalmady et al., 2021; Prakash et al., 2021; Ren et al., 2021; Wang et al., 2021; Weiss et al., 2021). For example, in CNN, there are convolution layers and fully-connected (FC) layers and in transfer learning one can transfer weights associated with convolution layers, FC layers, or both, then decide to freeze or fine-tune them. In SVM, one can transfer the kernel trained from one domain to the other domain. There was no transfer learning in methods other than CNN and SVM among the included studies. Table 5 shows different approaches used in studies in this review.

**Table 5:** Different transfer learning approaches.

**Transfer learning approach**	**Frequency**	**Percentage**
Kernel learning (KL)	8	13%
Freeze convolution and FC layers (FF)	12	19%
Freeze convolution layers and fine-tune FC layers (FT)	9	14%
Freeze convolution layers and randomly initialize FC layers (FI)	8	13%
Fine-tune convolution and FC layers (TT)	23	37%
Fine-tune convolution and initialize FC layers randomly (TI)	3	5%
All	63	100%

#### Kernel Learning

Kernel learning approaches are a category of algorithms for pattern analysis, mostly implemented in the context of SVM. Kernel learning finds general types of relationship in dataset to determine specific patterns. Kernel learning is also called instance-based learner because it can learn from specific training instance individually and update weights accordingly. Van Opbroek et al. (2019) proposed two different Kernel learning approaches to help SVM in image segmentation task. A method called multiple kernel learning was designed to minimize within-class distance and maximize between-class distance. Cheng et al. (2015) presented a kernel learning algorithm based on the multi-tasking Lasso to map samples from their space to the kernel space and then performed Lasso for selecting samples.

#### Freeze Convolution and FC Layers (FF)

Freezing all layers except the output layer was the third most common strategy. Freezing all layers implies that no training is required on those layers, lending this as the fastest method among others in the training step of the target domain. Al Vakli et al. (2018) used different strategies for their transfer learning in age classification problem. In one of their settings, all layers were transferred directly to the target dataset and frozen. Only the output layer of the network was trained. The performance of this strategy was 2% superior to training from scratch with much less training time. Dong et al. (2019) pre-trained AlexNet architecture on ImageNet dataset and then removed the output layer and freeze the rest of the model as feature extractor of the ADNI, as the target dataset. Hon and Khan (2017), Dai et al. (2019), Pham et al. (2019), Ebrahimighahnavieh et al. (2020), Kossen et al. (2021), Ocasio and Duong (2021), and Ramzan et al. (2020) utilized this approach in their study as well.

#### Freeze Convolution Layers and Fine-Tune FC Layers (FT)

Freezing convolution layers uses the CNN layers as feature extractor and then adds classifier or regressor layers above those features to decide the class or value of the output. Fine-tuning FC layers uses those layers and learned knowledge from another domain to our target domain. Therefore, FC layers are used as the classifier or regressor by initializing using pre-trained weights which make the training much faster and helps reach convergence much quicker. This was the most commonly used approach. Khan et al. (2019) developed layer-wise transfer learning to predict AD, MCI, and NC by fine tuning VGG 19 architectures. For the layer-wise transfer learning, five different settings were examined. Layers from 1 to 4, 1 to 8, 1 to 12, 1 to 16, and 0 were frozen for the first, second, third, fourth, and fifth settings, respectively. FC layers were fine-tuned in all settings. Optimal number of layers to be frozen depends on training set size. The larger the training data set, less layers are required. So, for larger datasets, only training the fully connected layers was enough. Regardless of differences in ImageNet and ADNI dataset, learned features from ImageNet are also useful for learning ADNI features and only the last layers which are related to specific tasks need to be fine-tuned.

Choi et al. (2020) developed a CNN algorithm to detect PD subjects with dementia. The model was trained on ADNI dataset and transferred to the Parkinson dataset. All convolution layers remained the same and only FC layers were fine-tuned. The area under the receiver operating curve (AUC) was 0.82 on the PD dataset while the performance on the source dataset was 0.81, which shows the knowledge was transferred appropriately. Al Vakli et al. (2018) implemented this approach in their settings and the performance improved from 84 to 91.2% of accuracy when compared with training from scratch. The FT approach outperformed the FF by 5.2% of accuracy which is a significant improvement. Han (2017), Li H et al. (2018), Wong et al. (2018), Zhou et al. (2018), Gao et al. (2019b), and Wee et al. (2019) examined this approach too.

#### Freeze Convolution Layers and Randomly Initialize FC Layers (FI)

In this FI approach, convolution layers act as a feature extractor without modifications. Other layers are initialized randomly, i.e., no knowledge is transferred from source domain to target domain for those layers. This approach is not as fast as FF and FT but tends to have a strong performance. Some studies used this method either because FC layers had to be modified in architecture used for the target dataset or classifiers different than the main algorithm were used, so that transferring weights in FC layers and output layer is not the case anymore. Oh et al. (2019) transferred convolution layers weights from an unsupervised autoencoder and added some classifier at the end of them to classify progressive MCI vs. normal control. Their results show that the fully trained CNN got 0.68 of accuracy, 0.75 of sensitivity, and 0.60 of specificity. These metrics for transfer fine-tuned CNN on the same task were 0.77, 0.81, and 0.74, respectively. The dataset was not balanced, which could be a reason why specificity is always lower in their implementation.

Saba et al. (2020) deployed transfer learning from VGG-19 pre-trained on ImageNet dataset to extract features from BRATS 2015, 2016, and 2017 datasets. Both BRATS 2015 and 2016 datasets include 220 high grade glioma (HGG) and 54 low grade glioma (LGG) in the training and 110 of HGG and LGG in testing phase. BRATS 2017 has 210 of HGG and 75 of LGG subjects. At the top of their architecture, different classifiers such as SVM, logistic regression, and K-nearest neighbor (KNN) were included. Their results show powerful performance, achieving dice similarity coefficient of 0.99 for BRATS 2015 and 2017, and 1.00 for BRATS 2016 dataset. Accuracy, specificity, and sensitivity of the algorithm was more than 0.99 for most cases.

Gao et al. (2019a) studied decoding behavior tasks using fMRI images. In this work, authors implemented several transfer learning algorithms and compared their results with the same algorithms but with training from scratch. AlexNet, ResNet, and Inception algorithms were implemented for both scenarios. For transfer learning, all convolution and pooling layers in the three algorithms were kept intact and few fully-connected layers? were added at the end. For the fully trained algorithms, the parameters were initialized using Gaussian distribution. Sensitivity, specificity, positive predictive value, negative predictive value, and accuracy was reported as performance metrics. Their results show transfer learning algorithms outperform fully-trained ones by more than 5% of accuracy on average. Other measures also show similar superior performance. Al Vakli et al. (2018), Jain et al. (2019), and Wang et al. (2019) also implemented this approach.

#### Fine-Tune Convolution and FC Layers (TT)

In this TT approach, all layers of CNN are initialized using pre-trained weights and all layers will be fine-tuned on the new dataset. Since convolution layers, especially first convolution layers, are associated with learning high level features such as lines, edges, and curves and last layers are more related to the task, it is more reasonable to perform training on FC layers than the convolution ones. Al Vakli et al. (2018) examined this approach and the best results came from this approach. Thomas et al. (2019) implemented this approach and obtained 92.43% of accuracy in classifying images based on brain activities. Different portions of the target dataset were tried and even with 1% of target dataset, the performance was 67.51% for transfer learning compared to 32.49% in full-training which is a considerable improvement. Amin et al. (2019) fine-tuned convolution and FC layers for AlexNet and GoogLeNet architectures pre-trained on ImageNet dataset. Several classifiers were added to the end of FC layers and obtained an accuracy of 88–100% for different classifiers such as KNN, Naïve Bayes, SVM, and Logistic Regression on top of AlexNet and GoogLeNet. Liang et al. (2018), Eitel et al. (2019), Khan et al. (2019), Oh et al. (2019), Puranik et al. (2019), Ramzan et al. (2020), Simon et al. (2019), Wu et al. (2019), Zhang et al. (2021), and Ocasio and Duong (2021) also utilized this approach and obtained competitive results.

#### Fine-Tune Convolution and Initialize FC Layers Randomly (TI)

In this approach, convolution layers are fine-tuned to have a better feature extractor when compared with the FI approach. Besides that, it is very similar to FI in the case of its applications. This approach was the least common approach. Wang et al. (2019) fine-tuned AlexNet as feature extractor and then added one new FC layer and randomly initialized the FC layer. At the end of their architecture, a SoftMax layer was applied. Al Vakli et al. (2018) and Qiu et al. (2018) were two other studies that implemented this approach.

### Neuroimaging Research Areas

Eleven different neuroimaging research areas were explored in the studies and among them AD related problems dominated the literature (see Table 6).

**Table 6:** Different research problem discussed by the literature.

**Research problem**	**Frequency**	**Percentage**
AD	29	58%
Brain mapping	8	16%
Age classification	2	4%
Brain tumor	3	6%
MS	1	2%
Obsessive-compulsive disorder	1	2%
Autism	1	2%
Arterial spin labeling	1	2%
Brain diseases	2	4%
Alcoholics detection	1	2%
Parkinson's disease	1	2%
**Total**	**50**	**100%**

Around 53% of studies attempted to offer a model that can classify MCI and normal controls from AD. Classification of AD, MCI, and normal controls was also studied extensively (Khan et al., 2019; Puranik et al., 2019; Li Y et al., 2021; Yang and Hong, 2021). Detection (Choi et al., 2020), Classification (Cheng et al., 2017), and autoencoder (Oh et al., 2019) methods were utilized to differentiate MCI from AD and/or normal controls. Other approaches include classification of normal control, early MCI, MCI, late MCI, and AD on fMRI images (Simon et al., 2019) and AD clinical score prediction and regression using CNN algorithm with transfer learning problem (Dong et al., 2019).

The fMRI images that show the brain activity, on a subset of Human Connectome Project (HCP)^1^ dataset which contains seven different behavior tasks were utilized for decoding behavioral tasks using (Gola et al., 2014; Gao et al., 2019a,b; Van Opbroek et al., 2019). Brain tumor diagnosis and segmentation of actual lesion symptoms using deep learning methods and transfer learning techniques were investigated (Liang et al., 2018; Amin et al., 2019; Saba et al., 2020). BRATS 2013, 2014, 2015, 2017 (Menze et al., 2015; Bakas et al., 2017, 2018) and ischemic stroke lesion segmentation 2018^2^ were the main datasets used for brain tumor segmentation and detection. Identification of alcoholism using transfer learning from AlexNet algorithm was studied by Wang et al. (Wang et al., 2019). As one of the first studies to implement CNN in this area, this study demonstrated that alcohol diminishes gray and white matter and that these effects can be captured using MRI images. Their dataset consists of 188 alcoholic and 191 non-alcoholic brain images. Among all transfer learning approaches implemented in this study, the setting with replacing just the last layer outperforms other settings that freeze less layers with around 97% on almost all metrics including sensitivity, specificity, precision, accuracy, and F1. Their results also show that data augmentation helped increase performance by 1–2%. Al Vakli et al. (2018) investigated transfer learning in age category classification and regression using resting state fMRI images. The source data set was a combination of functional connectivity dataset from publicly available datasets consisting of 368 fMRI from 200 subjects from three classes of young, middle age, and elderly age groups. The target dataset was collected in-house, consisting of 57 subjects (28 young and 29 elderly subjects).

Detection of autism spectrum disorder (ASD) subjects were explored using resting-state MRI images from Autism Brain Imaging Data Exchange (ABIDE) dataset (Di Martino et al., 2014). The CNN models in this study were pre-trained on the same dataset but with different tasks and then transferred to autism identification task in the ABIDE dataset (Li H et al., 2018). Choi et al. (2020) was the only study that utilized transfer learning in Parkinson's Disease. Eitel et al. (2019) implemented transfer learning to transfer knowledge gained from ADNI dataset to detect MS more efficiently. This study showed that CNN, without providing any information about MS related features, was able to obtain the same results as algorithms with handcrafted features from clinicians. In a study by Talo et al. (2019)s, normal brain and four brain diseases including degenerative, inflammatory, cerebrovascular, and neoplastic diseases were classified.

## Discussion

### Choice of Transfer Learning Approaches

Among the six transfer learning approaches, Kernel learning was only used in conjunction with traditional machine learning algorithms such as SVM. This method can be used when available data is very limited and using CNN algorithms is not possible. The main advantage of this method is that it requires little training time and resources, while also being easily interpretable, and this is another benefit of using SVM. However, since the performance of the SVM cannot match the performance of CNN architectures, utilizing this approach is fading away.

For CNN methods, freezing convolution and FC layers were used when the source and target dataset are similar and the task on both datasets are almost the same. For example, this approach can be utilized when a model is trained on an AD dataset (source) to classify the MRI images into binary classes of AD vs. normal control and weights are transferred to a classification model on another AD dataset (target) with the MRI images. Another application of this approach is in external validation where weights should not be updated. The advantage of this method is it requires zero training and therefore it is very fast and efficient. However, if the source and target datasets are very different from each other, other transfer learning approaches are preferred.

Freezing convolution layers and fine-tuning the FC layers can be used when the source and target datasets are different. This method uses the convolution layers as feature extractor. After extracting features from source dataset, it will be fixed for the target dataset and training can only be done on FC layers. However, the weights of the FC layers are transferred and fine-tuned on the target dataset to account for differences between both datasets. Since most CNN architectures such as VGG and ResNet have the majority of trainable parameters in FC layers, using this approach is not as fast as freezing all layers, but it usually achieves better performance because it fine-tunes the FC layers. Freezing convolution layers and fine-tuning FC layers is the second most successful approach. It needs less time and resources than fine-tuning all layers but, in some cases, would not result in the best results but still is quite competitive.

Freezing convolution layers and initializing the FC layers randomly is used when the model for the target dataset has the same convolution layers but different FC layers than the model for the source dataset. Studies included in our review changed the FC layers for a variety of design reasons such as minimizing the number of parameters, modifying the output layers, and modifying the number or size of FC layers. On the other hand, since the convolution layers are the same, studies generally preferred to use pretrained weights for the intact layers. This approach is slower than the FT approach but has different use cases.

When the source dataset and target datasets are very different from each other, such as ImageNet as source and ADNI as target dataset, fine tuning all layers including convolution and FC layers is the best approach. Here, the model (number and size of the convolution, pooling, FC layers) for both source and target datasets should be the same. Since this approach modifies the weights of all layers, it requires more time than other approaches. For CNN methods, fine-tuning all CNN layers demonstrated the best performance. This can be attributed to the flexibility to change the weights. If the best weights can be found by freezing all layers, this method can maintain current weights without modifying them. If the weights need to be tuned for a new domain, it can be easily trained on the target dataset and learn specific knowledge required for its specific task. However, the main drawback of this method is that it would take longer time and more computational resources than other methods but with slightly better performance results.

If the source and target datasets are different and the CNN architecture needs modifications in FC layers, then the best option would be fine-tuning convolution layers and initializing the new FC layers randomly. This method comes with highest training time among all transfer learning approaches, almost even close to full-training approach in some cases where the number of trainable parameters is much larger in FC layers than the convolution layers. However, the performance of this approach outperforms the full-training approach even in such cases.

Freezing all layers is the fastest approach and needs little resources and has acceptable results in some cases. Therefore, it is recommended to try freezing all layers at first, then try to fine-tune FC layers and finally attempt fine-tune convolution layers. If time and resources are not an issue, trying layer-wise would find the best setting for any specific problem. Table 7 summarizes strengths and limitations for different transfer learning approaches.

**Table 7:** Strength and limitations of transfer learning approaches.

**TL method**	**Strengths**	**Limitations**
KL	Needs little computational resources and time.	Cannot be implemented on CNN algorithms.
FF	Does not need training or fine-tuning. Also, does not need large computational resources.	Cannot generalize well on very different source and target datasets. Fewer applications.
FT	Convolution layers act as feature extractor and do not need to be trained again. Diverse applications. Faster than most methods other than FF. Second in performance after TT.	Needs more hardware resources than FF because of fine-tuning of FC layers. May not be as successful as TT if source and target datasets are very different.
FI	Convolution layers act as feature extractor and do not need to be trained again. Diverse applications. Faster than most methods other than FF.	Needs more resources for FC layers to be trained from scratch. Slow.
TT	Best performance among all methods. Very flexible.	Needs more resources for fine-tuning convolution and FC layers. Slow.
TI	Strong performance, almost as good as FT. Much more flexible than other methods, including TT.	Slowest. Needs much more resources than other methods.

There were at least six transfer learning approaches utilized in neuroimaging studies. In almost all studies, transfer learning improved the performance metrics such as accuracy, AUC, specificity, and sensitivity. Therefore, it is recommended to deploy this strategy while working with neuroimaging data, especially, when the dataset is limited. However, finding the best approach among all transfer learning approaches could be a little challenging. Based on this review, the performance of transfer learning algorithms, for example in AD classification, are very different from one study to another (accuracy between 80 and 100%) even with the same dataset. This could be because of different combinations of subjects used for training and testing, or different hyper-parameters. Applying hyper-parameter tuning and cross validation techniques would help to address these issues.

Lack of a large-scale annotated datasets was another unique challenge in medical imaging, especially neuroimaging. Large general-purpose datasets such as ImageNet has helped researchers to not only design successful algorithms for general image processing but also helped design better models for medical image processing. Developing large datasets specific to medical imaging with consideration to attributes such as 3D or 4D data, and multimodal data, will result in designing much better algorithms. In addition, competitions for algorithm development competition challenges in neuroimaging, counterpart with ImageNet challenge in general image processing, would help to have more successful algorithms to be developed by researchers.

### Open Challenges and Future Trends

One of the issues with imaging datasets is that, in most cases, source and target datasets are different from each other in terms of size and feature characteristics. If these input sizes are different, the convolution and FC layers parameters' shape and size would be different too. Transfer learning is not possible unless some modification is done regarding the sizes. In our review, we found one single strategy used by researchers (Hon and Khan, 2017; Qiu et al., 2018; Eitel et al., 2019; Jain et al., 2019; Khan et al., 2019; Simon et al., 2019; Ramzan et al., 2020) to tackle this issue and that was resizing the target domain to match the size of the pre-trained architecture. While this was a successful strategy, we would lose useful information when resizing a medical image to a lower size. We believe developing new strategies that does not need resizing images will be a interesting future direction.

Interestingly, one topic that was overlooked by all studies is that no transfer learning approaches that can transfer knowledge from 2D to 3D dataset were explored. All studies that had 2D dataset as the source dataset, implemented 2D algorithms for the target dataset even if their target dataset was 3D. A related open challenge is that there is no publicly available pretrained 3D architecture that can be used directly on transfer learning of 3D CNN architectures. Providing pretrained 3D architecture, trained on neuroimaging data would be a promising future research direction. Another challenge that can contribute significantly to applying transfer learning on 3D data is to design an algorithm to transfer 2D knowledge (i.e., pre-trained weights on 2D data) into 3D space. One potential solution could be concatenating of different kernels to form 3D kernels. Such more effective and elaborated approaches can also be explored.

Another gap we found in the current literature is that transfer learning to fMRI is rare, where there are four dimensions: depth, width, height, and time. For spatial features, CNN is typically used. For the temporal dimension, a time series approach such as LSTM is utilized. Here, the challenge would be how to transfer weights into both CNN and LSTM. One approach can be using the same transfer learning for the CNN part and initialize the LSTM weights randomly. However, if a mixed model (i.e., CNN+LSTM) can be trained on a source dataset, then the LSTM weights can be transferred too. This is also a potential research direction that we hope that the community will explore in the near future.

## Conclusion

Transfer learning is one of the successful strategies when processing small-scale datasets such as neuroimaging datasets. It is especially necessary and appropriate to implement transfer learning when the target dataset is very small and using existing models results in under-fitting. Transfer learning helps to learn knowledge from a source dataset and use that knowledge to solve related problems in target datasets. In this review of literature related to transfer learning algorithms in the neuroimaging, we identified and summarized different source and target datasets, imaging modalities, research problems, and transfer learning approaches. Our results show that implementing transfer learning helped improve the performance of algorithms for neuroimaging applications in almost all cases. Transfer learning algorithms were able to provide better results than fully-trained algorithms using less time and resources. Among all transfer learning approaches, fine-tuning all layers tends to have the best performance. Furthermore, using non-neuroimaging datasets, even general-purposes imaging datasets such as ImageNet, helps with improving model performance.

## Author Contributions

ZA performed the review and wrote the first draft of the manuscript. VS conceptualized the idea, assisted with the review process, and provided overall supervision. Both authors reviewed, made critical edits to the manuscript, and approved the submitted version.

## Funding

This work was supported in part by the National Science Foundation Under Grant #1838745.

## Conflict of Interest

The authors declare that the research was conducted in the absence of any commercial or financial relationships that could be construed as a potential conflict of interest.

## Publisher's Note

All claims expressed in this article are solely those of the authors and do not necessarily represent those of their affiliated organizations, or those of the publisher, the editors and the reviewers. Any product that may be evaluated in this article, or claim that may be made by its manufacturer, is not guaranteed or endorsed by the publisher.

## Footnotes

^1^Human Connectome Project | Mapping the Human Brain Connectivity.

^2^ISLES: Ischemic Stroke Lesion Segmentation Challenge 2018.
